# Cancer Research

**Published:** 1904-10-29

**Authors:** 


### CANCER RESEARCH.

A new line of investigation in the pathology of cancer appears to have been opened up by the observations of Farmer, Moore, and Walker[1] on the process of karyokinesis in the cells of malignant tumours. In the division of cells in normal tissues a uniform series of changes takes place in the nucleus. Each nucleus exhibits a definite number of chromosomes, which is constant for each species of the higher animals. Prior to the division of the cell the chromosomes assume the appearances of delicate rods or V-shaped bodies, which lie in the equatorial plane of the nuclear spindle with their apices directed towards the axis. Each of the rods then splits lengthwise into two, one half going to each pole of the cell which then divides, with the result that each of the new cells formed by the division has the same number of chromosomes as the parent cell. There is one exception to this, and that is in the case of gametogenic (germinal or reproductive) tissue. In these cells the chromosomes form bodies of irregular shape, are arranged longitudinally on the spindle, divide transversely, and are present in only half the number proper to the species. The result is that when fusion of two of these sexual cells (ovum and spermatozoon) takes place, the resulting cell (fertilised ovum) has the full complement of chromosomes proper to the species. A remarkable fact in connection with gametogenic tissue is that it sometimes acts as a parasite on the parental tissues; as illustrating this fact, is quoted the behaviour of the embryo sac in the ovule of a flowering plant which acts in a parasitic manner, sometimes invading and destroying the tissues of the parent organism. Now these observers have discovered that a heterotype mode of nuclear division, very similar to that observed in normal reproductive tissue, takes place in the cells of malignant tumours. They state that they have not failed to find evidence of this heterotype mitosis in any cancerous growth examined; the more rapid the growth the more readily may it be observed. No example of this process has been detected in normal tissues or in benign tumours. They consider that the malignant character of sarcomata and carcinomata may be attributed to these intracellular phenomena. The discovery may also prove of practical utility in the differentiation of innocent and malignant growths.

Bashford and Murray[2] in a report, as the result of investigations carried out in connection with the Cancer Research Fund, fully confirm these observations, both in human and animal cancer. In the same report they deal with the zoological distribution and transmissibility of cancer. They have received numerous specimens of malignant growths from various domestic animals, particulars of which are enumerated in a table. Most of the specimens were obtained from the dog, the cow, and the horse, several from fishes. No case of cancer in an invertebrate animal has so far been recorded. The microscopic characters of these growths were identical in every essential feature with the same growths in man. These facts as to the prevalence of cancer among the lower animals are interesting when considered in connection with the theories which assign articles of diet, alcohol, syphilis, and the developments of civilisation as the causes of cancer. The transmission of cancer from man to animals, or from one animal to another of a different species, has never been successfully performed. Successful transplantations have been made of growths from one animal to another of the same species. They believe that transplantation is identical with the process of metastasis, and that it is in no sense an infection, the tissues of the host in no way participating in the formation of the new parenchyma. The widespread zoological distribution of cancer indicates that the cause is to be sought in a disturbance of those phenomena of reproduction and cell life which are common to the forms in which it occurs.

[1] Brit. Med. Jour., Dec. 26, 1903. [2] Lancet, Feb. 13, 1904.

*(To be continued.)*

